# Experiences of family members who have a relative diagnosed with borderline personality disorder

**DOI:** 10.4102/curationis.v41i1.1892

**Published:** 2018-10-03

**Authors:** Marcia L. Kay, Marie Poggenpoel, Chris P. Myburgh, Charlene Downing

**Affiliations:** 1 Department of Nursing, University of Johannesburg, South Africa; 2 Department of Educational Psychology, University of Johannesburg, South Africa

## Abstract

**Background:**

While working in a psychiatric hospital, the researcher observed the beneficial effects of the mindfulness programme offered to patients diagnosed with borderline personality disorder (BPD). Little support was offered to family members who attempted to manage their relatives diagnosed with BPD. The family members often experienced stress, depression, grief and isolation.

**Objectives:**

The purpose of this research was to explore and describe the experiences of family members who have a relative diagnosed with BPD.

**Method:**

A qualitative, exploratory, descriptive and contextual study design was used. A purposive sample of family members aged between 24 and 74 years was made. Data were collected by conducting eight in-depth, phenomenological interviews, and field notes were taken. The interviews focused on the central question, ‘Tell me your experience of having a relative diagnosed with BPD’. Tesch’s method for data analysis was used, and an independent coder analysed the data and met with the researcher for a consensus discussion of the results. Measures to ensure trustworthiness were applied and ethical principles were adhered to.

**Results:**

Four themes were identified. In theme one, family members described their relative diagnosed with BPD as experiencing emotional, behavioural, interpersonal and self-dysregulation. As a result, family members expressed negative feelings towards their relatives and experienced social humiliation, financial strain and marital discord. In theme two, they experienced a desire to move forward and improve their mental health. In theme three, family members experienced a challenging process of adaptation and coping. In theme four, family members experienced a quest for harmony and integration.

**Conclusion:**

The results indicated that family members experienced disempowerment because they lacked knowledge about their relative’s BPD. More knowledge about BPD, the understanding and implication of the diagnosis, treatment and care are needed from mental health care professionals and service providers.

## Introduction

Borderline personality disorder (BPD) is a complex and challenging mental health condition for the person and the family members who support them (Lawn & McMahon 2015:234–243). Patients diagnosed with BPD have been seen flooding mental health facilities, in addition to receiving treatment from their own general practitioners. These patients were reported to be challenging to manage and presented with a pervasive pattern of instability related to emotional dysregulation and impulsivity. This dysregulation is characterised by unstable, intense interpersonal relationships, affect instability and low self-image. Patients often experience periods of psychosis in stressful situations. Self-mutilation and substance abuse are common (Kaplan & Saddock 2015:500). Borderline personality disorder affects approximately 2% of the general population; however, the Diagnostic and Statistical Manual of Mental Disorders-V (American Psychiatric Association [APA] 2013:665) states that 1.6%, and as high as 5.9%, of the general population could meet the criteria for this diagnosis. Borderline personality disorder has been considered a chronic, mostly untreatable disorder. Psychotherapeutic treatment of people with BPD is one of the most significant challenges confronting mental health professionals today. Few family-based education programmes exist that have been evaluated for family members of BPD patients.

The role of family members caring for individuals diagnosed with mental illness has expanded (World Health Organization 2017). Family members experience significant distress related to the relative diagnosed with BPD’s problems (Goodman et al. 2011:59–74). The majority of family members report difficulties in caring for their relative diagnosed with a mental illness (Weimand et al. 2010:804–815). Family members feel overwhelmed, unable to cope and exhausted by the stress (Bademli & Duman 2016:329–333). The burden experienced by family members with a relative diagnosed with BPD has been noted to be greater than those with other mental disorders (Bailey & Grenyer 2013:248–258, 2014:796–809). Sansone and Sansone (2013:39–43) state that mental health clinicians are not immune to negative attitudes towards patients with BPD. Stigma is also very common in BPD and research studies have indicated that psychiatrists hold negative attitudes towards patients with BPD (Bodner et al. 2015:2). Family members of individuals diagnosed with BPD sometimes experience challenges and discrimination when attempting to engage with health services. They often feel devalued, unsupported and kept uninformed of the treatment process (Bauer et al. 2012:956–971; Dunne & Rogers 2013:643–648). This lack of support from mental health clinicians creates a greater burden on family members with relatives diagnosed with BPD. The value in this study was the development of understanding on family members’ experiences of having relatives diagnosed with BPD, and recommendations to assist these family members.

## Problem statement

The researcher worked in a psychotherapy unit in a psychiatric hospital in Gauteng. The majority of patients were diagnosed with BPD. These patients were admitted and treated with various modalities including psychopharmacology, individual therapy, group therapy and dialectical behaviour therapy, which includes mindfulness. Little was offered to the family members in terms of education, except for a twice-weekly support group. Owing to the complexity of the illness, most families have little knowledge of the treatment programme undergone by their relatives while in the unit. Although patients gain skills from the programme, many return to repeat the programme, as little support was given to the family members to manage their relatives diagnosed with BPD upon discharge. Instead of being a resource to the relative diagnosed with BPD, the family members were often seen as having a negative effect on their relative’s behaviour. The knowledge gap identified in psychiatric nursing was that there was little knowledge regarding support available for family members, and family members’ experiences of having relatives diagnosed with BPD.

The research question was the following: ‘What was the experience of family members who have a relative diagnosed with BPD?’

## Research purpose

The purpose of the research was to explore and describe the experiences of family members who have a relative diagnosed with BPD.

## Definition of key concepts

### Experiences

Experiences refer to individuals’ descriptions of meaning regarding a phenomenon (Creswell 2014:56–57). In this study, experiences refer to the experiences of family members who have a relative diagnosed with BPD.

### Family members

Family members are persons who belong to a particular family (Oxford Dictionaries 2018b:n.p.). In this study, the term ‘family’ refers to the family members who have a relative diagnosed with BPD.

### Borderline personality disorder

Borderline personality disorder is a severe psychiatric disorder with a pattern of instability in interpersonal relationships, self-image, affects and marked impulsivity (APA 2013:636). In this study, relatives are diagnosed with BPD.

### Diagnosis

Diagnosis is the identification of the nature of an illness, or a problem by examination of the symptoms, and the distinctive characterisation (Oxford Dictionaries 2018a:n.p.). In this study, relatives are diagnosed with BPD.

## Research design and method

A qualitative, exploratory descriptive and contextual design was used in this study (Burns & Grove 2013:23), adopting a phenomenological approach. Phenomenological studies examine human experiences through the descriptions provided by the people involved (Creswell 2014:56).

### Population and sampling

The target population consisted of family members who had a relative diagnosed with BPD. Purposive sampling was used (Houser 2012:424). The criteria for inclusion were participants who were family members with a relative diagnosed with BPD, who were above 18 years of age and who could speak English. The participants were requested to sign a consent form for the interview, as well as for the audio-recording of the interview.

### Data collection

A pilot study consisting of two interviews was carried out with no difficulties experienced. Then, in-depth phenomenological interviews were conducted with one member of each of the eight families who have a relative diagnosed with BPD.

Open-ended questions were asked to all the participants. The central question asked to the participants was the following: ‘Tell me your experience of having a relative diagnosed with BPD’.

Data saturation was reached when no new themes or categories emerged from the in-depth phenomenological interviews (Gray, Grove & Sutherland 2017:254).

Field notes were used as a data collection method and were taken during the interviews. These field notes consisted of personal, methodological, observational and theoretical field notes (Gray et al. 2017:256–257; LoBiondo-Wood & Haber 2014:276; Polit & Beck 2017:22).

### Data analysis

The recorded interviews and field notes were transcribed and analysed using Tesch’s descriptive analysis technique of open coding (Creswell 2013:155–156). The results of the analysis were discussed with an independent coder who had a PhD in psychiatric nursing and was experienced in qualitative research. Then, a consensus was reached on the themes of the results. The results were supported by the literature.

## Trustworthiness

Guba’s model of trustworthiness was applied in this study. The four criteria of trustworthiness were utilised. These were credibility, transferability, dependability and confirmability (De Vos et al. 2011:419–420). Credibility includes prolonged involvement in the area of study. The researcher spent time with the participants in the psychotherapy unit, which offered the opportunity for extensive exposure to the environment. Triangulation of data took place through interviews, observation and field notes. Member checking was achieved, as the participants were provided with the results of the study to validate the correct interpretation of the interviews. An expert with a PhD in psychiatric nursing acted as an independent coder.

Transferability refers to the extent to which the findings of the inquiry have applicability in other contexts, or with other participants (Lincoln & Guba 1985:290). The demographics of the participants were described in depth. A dense description of the results with direct, supporting quotations from the participants was given. Dependability is the ability of an inquiry to yield the same result with repeated use of the same participants in the same context. A dense description of the research methodology was provided.

Confirmability establishes a degree to which the results of enquiry are determined by the participants and the conditions of the inquiry, and not by the biases or motivations of the researcher (De Vos et al. 2011:419–420). A chain of evidence was provided for the whole research process. The interview transcripts, audio-tapes and field notes were kept for this purpose. An independent coder carried out a confirmability audit, and the researcher and independent coder were able to reach consensus regarding the confirmability of the data.

## Ethical considerations

Approval from the ethics committees was obtained from the universities’ Faculties of Health Sciences Academic Ethics Committee (reference numbers AEC.01-552014 and M140657). The researcher applied the following four ethical principles according to Dhai and McQuoid-Mason (2011:13–14): autonomy, non-maleficence, beneficence and justice. Autonomy means that the researcher showed respect for the participants; family members made their own decisions to participate. Consent was obtained to carry out the study. Confidentiality was ensured, as data that identified the participants were not disclosed. The sum of potential benefits to a participant and the knowledge gained should outweigh the risk of harm (Kvale & Brinkmann 2014:68–72). Participants were not put at risk in this study.

Non-maleficence means that a person must not cause harm, such as conducting research without giving the subjects the permitted full information or protection. Debriefing sessions were available if required. Beneficence implies acting for the good of the participants (Gray et al. 2017:163–168). The researcher discussed that there were no financial benefits or harm anticipated for participants. The sharing of their lived experiences benefitted other people who were in the same situation and added to the scientific body of knowledge. The researcher managed the interviews appropriately to prevent any discomfort or harm by asking applicable questions, monitoring and observing for any sign of distress (Gray et al. 2017:163–168). Justice relates to fairness in the selection of people involved and distribution of the burdens and benefits of the research. Participants were selected according to the relevant criteria of the study.

## Findings

A description of the demographics of the participants and themes of family members’ experiences of a relative diagnosed with BPD follows.

### Description of the demographics of the participants

The participants who were interviewed consisted of three mothers, two of whom were 50 years old and one was 74 years old; one father, who was 50 years old; two husbands, who were 36 and 44 years old; one uncle, who was 56 years old; and one daughter who was 24 years old. The age range thus varied between 24 and 74 years. Table 1 presents the demographic data of the eight in-depth phenomenological interviews with family members as participants.

**TABLE 1:** Demographic data of family members as participants.

Interviews	Patient data		Family member’s relationship with the patient	Family member’s age in years	Race	Language
	Gender	Age in years				
Interview 1	Female	21	Mother	50	White	Afrikaans
Interview 2	Female	23	Father	52	Indian	English
Interview 3	Female	36	Husband	36	White	Afrikaans
Interview 4	Male	29	Uncle	56	White	English/Greek
Interview 5	Male	43	Mother	74	White	English/French
Interview 6	Female	43	Husband	44	White	Afrikaans
Interview 7	Female	45	Daughter	24	White	Afrikaans
Interview 8	Male	19	Mother	50	White	Afrikaans

*Source*: Kay, M.L. (2017). A model for the facilitation of mental health of family members who have a relative diagnosed with Borderline Personality Disorder in a psychiatric inpatient unit. D Cur Psychiatric Nursing Thesis, University of Johannesburg, Johannesburg. (p. 67)

#### Findings of the interviews: Themes of family members’ experiences of a relative diagnosed with borderline personality disorder

Four themes were identified from the data. Table 2 presents the findings of the interviews.

**TABLE 2:** Family members’ experiences of a relative diagnosed with borderline personality disorder.

Theme	Description
Theme 1	Family members experienced their relatives diagnosed with BPD as displaying emotional, cognitive, behavioural, interpersonal and self-dysregulation.
Theme 2	Family members’ intra- and interpersonal experiences of relatives diagnosed with BPD. This included negative emotional responses towards their relatives diagnosed with BPD owing to social humiliation, financial strain and marital conflict.
Theme 3	Family members experienced a challenging process of adaptation and coping.
Theme 4	Family members experienced a quest for harmony and integration.

*Source*: Adapted from Kay, M.L. (2017). A model for the facilitation of mental health of family members who have a relative diagnosed with Borderline Personality Disorder in a psychiatric inpatient unit. D Cur Psychiatric Nursing thesis, University of Johannesburg, Johannesburg. (pp. 68–69)

BPD, borderline personality disorder.

#### Theme 1: Family members experienced their relatives diagnosed with borderline personality disorder as displaying emotional, cognitive, behavioural, interpersonal and self-dysregulation

Chaos and family disorganisation were experienced as the result of relatives diagnosed with BPD displaying dysregulation in the family members’ home environment Kay, 2017:69-80).

Emotional dysregulation of the relative diagnosed with BPD was identified in the following quotations by participants:

‘She had anger outbursts, had threatened that she could murder me. My wife also went on the internet, shopping for sex and said, “Anytime is fair game”. She walked down naked at a party.’ (Participant 3, male, 36 years, husband)

‘He has needy ways and when confronted, gets upset, breaks into tears, as he wants to be perfect. He doesn’t want to be left alone.’ (Participant 4, male, 56 years, uncle)

Emotional dysregulation of relatives diagnosed with BPD caused difficulty in effectively managing their emotions. Cognitive dysregulation refers to relatives diagnosed with BPD who experienced episodes of dissociation. This study identified that relatives diagnosed with BPD were experienced by their family members as extremely sensitive to criticism, judgement and they felt unsupported by their family, as seen in the quotations below:

‘She is stubborn and dogmatic. When she left SANCA, she wrote a letter stating that her father didn’t love her, despite him being in the delivery room when she had her baby. This was out of love for his daughter.’ (Participant 1, female, 50 years, mother)

‘He is often suspicious, particularly in groups or crowds, so much that he imagines that others are talking about him. He will insist that his way, is the right way.’ (Participant 4, male, 56 years, uncle)

Behavioural dysregulation was seen when the relatives diagnosed with BPD felt overwhelmed by situations. They acted impulsively to regulate their emotions which resulted in destructive behaviours such as alcohol and drug abuse, cutting, unsafe sexual practices and suicidality. An inappropriate response could trigger a relative to act out, resulting in a potentially life-threatening incident. Supporting quotations from participants follow:

‘She was in the Eating Disorder Unit. She was a bulimic and was purging. I was surprised that she started to take drugs and cut herself; also to have tattoos. I never knew that she was very ill.’ (Participant 2, male, 52 years, father)

‘My experience started when my wife was scratching (self-mutilating) and later cut her wrist. She was overwhelmed by stress. She had relapses and tried to overdose on medication.’ (Participant 6, male, 44 years, husband)

Interpersonal dysregulation by relatives diagnosed with BPD often led to mood-dependent relationships. Family members were unaware of the unpredictable behaviours that their relatives may experience. These behaviours led to confusion and the destruction of relationships between the relatives and family members. These experiences were expressed in the following quotations by the participants:

‘She has chaos in her relationships. We couldn’t force her to do things. She pressurised her father, and was determined to get what she wants. The challenges caused a lot of unhappiness and fights.’ (Participant 1, female, 50 years, mother)

‘I couldn’t leave her on her own. I think it is quite harsh the things she says to me. I think she’s caught up in her own pain. It’s really tough for me. I need someone to say, “I’m not bad for her”.’ (Participant 3, male, 36 years, husband)

Relatives diagnosed with BPD who experienced chaos in their relationships had difficulty managing and maintaining relationships.

Self-dysregulation by relatives diagnosed with BPD was evident from the interviews conducted with family members. Relatives diagnosed with BPD were seen as unable to acknowledge any good within themselves. They lacked an ability to develop a consistent sense of themselves. They felt different in different places with different people. They experienced feelings of emptiness which resulted in harmful behaviour and inappropriate relationships. Family members lacked the ability to help. Quotations from the participants indicated:

‘She talks about how bad she is feeling. She becomes withdrawn, says negative things to me. I think the problem is within herself. She still feels stuck. She would ask me, “How is my love going to make her feel better?”’ (Participant 3, male, 36 years, husband)

‘He feels he is a burden and has no friends, feels others are talking about him. He has difficulty to separate his and others’ issues. He blames himself, internalises his feelings, which leads to his self-mutilation.’ (Participant 4, male, 56 years, uncle)

#### Theme 2: Family members’ intra- and interpersonal experiences of relatives diagnosed with borderline personality disorder

Family members experienced negative feelings towards their relatives diagnosed with BPD (Kay 2017:81-89). These feelings included despair, sadness and regret, humiliation, guilt and shame, financial challenges and marital conflict.

Family members experienced despair owing to the interpersonal conflict and crises of the relatives diagnosed with BPD. The pressure was put on the relationships of family members. Relatives criticised their family members and rejected their concerns, as seen in the quotations by participants:

‘She wanted to pack, go back to hospital. I said that I was sorry that she felt that way, that I am also feeling sad. I’ve come to a place where I feel absolutely worthless, it feels like I am walking on eggshells.’ (Participant 3, male, 36 years, husband)

‘It was a big shock for me. My first concern must be my family. If I don’t work on this, I’ll be attending hospital myself. It feels to me that I am half-dead already.’ (Participant 6, male, 44 years, husband)

Family members also experienced sadness and regret with relatives diagnosed with BPD. In the quotations that follow, the participants who were partners stated their determination to work on their relationships:

‘One day we will be over seventy, and we need to know she can stand on her own two feet. She must get healthy.’ (Participant 1, female, 50 years, mother)

‘I keep trying to be consistent. She can’t understand that I’m feeling down, but I’m also feeling sad.’ (Participant 3, male, 36 years, husband)

Relatives diagnosed with BPD frequently acted impulsively, resulting in destructive and inappropriate behaviour. Family members felt judged by their extended family and felt that they were viewed as ineffective at handling their relative’s behaviours, as identified in the following quotation:

‘It causes a lot of family arguments. She was not willing to share her room. She obviously can’t see how she affects us. She loses the ability to put herself in other people’s shoes.’ (Participant 7, female, 24 years, daughter)

Financial strain created by their relative’s inappropriate behaviours was evident. Family members paid for medical treatment to manage the disorder, drug or alcohol rehabilitation, inpatient psychiatric treatment, along with the damages incurred to themselves and others. Quotations from participants indicated:

‘I was under pressure with the finances. When I didn’t disclose this, she saw this as lying to her. Last year, I was spending a lot of time with her. It happened that I lost the contract. Had to get another job.’ (Participant 3, male, 36 years, husband)

‘I don’t want him to think I am a controlling mother, but he relies on me for money. We can’t carry on in this manner. He’s having to rely on me. His wheels fell apart.’ (Participant 5, female, 74 years, mother)

Family members experienced marital discord owing to extra conflict and ongoing stressful challenges created at home by relatives diagnosed with BPD. Some participants revealed:

‘It causes a lot of family arguments when she doesn’t listen. I had to visit her in hospital every day. I didn’t have a life and this put a strain on my relationship with my husband.’ (Participant 1, female, 50 years, mother)

‘I was under pressure with the finances, and I disclosed this. She saw this as lying to her. The relationship may end over my blindness, I have tolerated a lot of things, but I continue to do my best to be supportive.’ (Participant 3, male, 36 years, husband)

#### Theme 3: Family members experienced a challenging process of adaptation and coping

Family quarrels and fights because of relatives diagnosed with BPD led to poor, ineffective coping strategies (Kay 2017: 90-94). This resulted in a feeling of disempowerment of family members as they lacked the ability to control the circumstances in their family life. Health services were available but were limited and understaffed. There was limited knowledge and information available to educate the family members to cope effectively with relatives diagnosed with BPD. Quotations from the participants indicated:

‘I’m not sure whether I should ask questions about what he’s done. I blame myself because I was strict with him. My daughter says I must go to Tough Love.’ (Participant 5, female, 74 years, mother)

‘If I can’t support myself, how can I support my children? I have to be both mum and dad. I don’t want extra stress in my life. I need to help her control her stress, as I’m fearful of the consequences.’ (Participant 6, male, 44 years, husband)

Ineffective coping strategies were related to a lack of communication skills and knowledge by family members of relatives diagnosed with BPD. The lack of awareness of BPD resulted in the health system’s failure to meet the needs of their patients. Some medical practitioners refused to treat patients diagnosed with BPD owing to their lack of knowledge. This was illustrated in the following interviews:

‘I don’t know how to nurture her in the right direction. I never knew she was very ill, taking drugs and hurting herself. She can’t run away from real issues. I was angry with myself. I never communicated with my child. Something has to change. I just wasn’t there for her.’ (Participant 2, male, 52 years, father)

‘I used to tiptoe all around him. As parents, we are often anxious to open the communication, and then we get no response. We must wait for the right time to have a meaningful conversation. I was focusing on the drug addiction and depression. I didn’t know that everything was working together.’ (Participant 8, female, 50 years, mother)

Ineffective coping skills by family members with relatives diagnosed with BPD were often related to a lack of knowledge. This prevented family members from making appropriate choices in assisting their relatives diagnosed with BPD.

‘I only realised after I’d married her that she had BPD. If she sleeps with someone else, does that mean she doesn’t care about me? I need help to set limits and cope with her needs.’ (Participant 3, male, 36 years, husband)

‘I’m not sure if my wife wants to have more knowledge, but I think it’s worth an attempt. Some of the questions asked by other family members come from a lack of experience. They are not sure how to deal with their relatives and why they react the way they do.’ (Participant 4, male, 56 years, uncle)

#### Theme 4: Family members experienced a quest for harmony and integration

Family members wanted to change their approach to caring for their relatives diagnosed with BPD and become reconnected. They wanted to be self-determined, self-empowered and able to trust, care and support themselves. Family members were motivated to care for their relatives diagnosed with BPD in an empowered way (Kay 2017: 94-104). The following quotations from participants stated:

‘I have got a lot of experience of mental illness. I notice a pattern of behaviour with my nephew, and I take action to calm him. I don’t have a choice. It’s my family, we’re very family orientated.’ (Participant 4, male, 56 years, uncle)

‘I gave socks and shoes so that he can start running and enjoy it. I want him to let go of all his baggage.’ (Participant 5, female, 74 years, mother)

Family members were eager to develop a renewed sense of trust and support. They were willing to develop new skills and accept the community’s support. They identified what was helpful, as seen in the following quotation:

‘I’m in a much better place now. I have a list of telephone numbers now. I started to “phone other people just to talk to them”.’ (Participant 3, male, 36 years, husband)

The family members stated that they needed to be self-caring in order to be adequately skilled to provide care for their relative diagnosed with BPD. Quotations from the participants reflected:

‘I would like to complete the parenting programme, to show me how to help her.’ (Participant 1, female, 50 years, mother)

‘I need help to set limits, to cope with situations and to have appropriate responses when she loses her temper.’ (Participant 3, male, 36 years, husband)

Family members experienced the need for professional and community support as illustrated in the following quotations:

‘I would like to continue to get feedback from the outpatient group. There is a lot of misinformation.’ (Participant 2, male, 52 years, father)

‘People keep quiet and the knowledge is not readily available. There has been no information available for the partner’s side.’ (Participant 6, male, 44 years, husband)

## Discussion of findings

### Family members experienced their relatives diagnosed with borderline personality disorder as displaying emotional, cognitive, behavioural, interpersonal and self-dysregulation

Chaos and family disorganisation was seen in family members’ home environment because of the display of dysregulation by relatives diagnosed with BPD. In this study, the emotional dysregulation displayed by relatives diagnosed with BPD caused difficulty in effectively managing their emotions. There is an emotional response to the conflict in relationships and difficulty in naming or fully experiencing emotions (Aguirre & Galen 2013:23). Fruzzetti et al. (2009:272–284) claim that emotional dysregulation occurs when someone is unable to change or accept parts of their emotional experience. They experience high, negative emotional arousal, which interferes with their ability to function.

Cognitive dysregulation displayed by relatives diagnosed with BPD led to episodes of experienced dissociation. The study identified that relatives were extremely sensitive to criticism, judgement and felt unsupported by their family members. Porr (2010:55) states that discussions that seem logical to the family member are often emotionally interpreted as judgemental or blaming by their relatives diagnosed with BPD.

Behavioural dysregulation was seen when the relatives diagnosed with BPD felt overwhelmed by situations. They acted impulsively to regulate their emotions, which resulted in destructive behaviours such as alcohol and drug abuse, cutting, unsafe sexual practices and suicidality. An inappropriate response could trigger a relative to act out, resulting in a potentially life-threatening incident. Impulsivity is a key aspect of BPD. The most dangerous behaviours are self-injury, drug use, unprotected sex with unknown partners and reckless driving (Aguirre & Galen 2013:13). The individual uses these behaviours initially as a way to regulate and reduce their intense emotions (Brooke & Horn 2010:113–128; Feigenbaum 2010:115–134).

Interpersonal dysregulation often leads to mood-dependent relationships. Family members are unaware of the unpredictable behaviours that their relatives may experience. These behaviours lead to confusion and the destruction of relationships between the relatives and family members. Relatives diagnosed with BPD fear abandonment by the important people in their lives (Aguirre & Galen 2013:23). Their experience of disagreement, ambivalence, anger, sadness and emptiness seems to mark their interpersonal experience in all of their relationships (Stepp et al. 2009:484–491). People who experience chaos in their relationships have difficulty managing and maintaining relationships.

Self-dysregulation displayed by relatives diagnosed with BPD was seen as them being unable to acknowledge any good within themselves. Relatives diagnosed with BPD lacked the ability to develop a consistent sense of themselves. They felt different in different places with different people. They experienced feelings of emptiness which resulted in harmful behaviour and inappropriate relationships. Family members lacked the ability to help these relatives diagnosed with BPD.

Self-dysregulation displayed by relatives diagnosed with BPD consisted of identity disturbances, such as the lack of self-identity and a low self-acceptance. Crowell, Beauchaine and Linehan (2009:495–510) and Aguirre and Galen (2013:23) state that relatives diagnosed with BPD have difficulty experiencing themselves as integrated people and struggle to define a sense of themselves. They experience changes in their values, preferences, identity and self-image.

### Family members’ intra- and interpersonal experiences of relatives diagnosed with borderline personality disorder

Family members experienced negative feelings towards their relatives diagnosed with BPD. These feelings included despair, sadness and regret, humiliation, guilt and shame, financial challengers and marital conflict.

Despair was experienced owing to the interpersonal conflict and crises with the relatives diagnosed with BPD. The pressure was put on the relationships of family members. Relatives diagnosed with BPD criticised their family members and rejected their concerns.

Family members experienced family strife and rejection (Bauer et al. 2012:956–971; Bowen 2013:491–498; Wedig, Franenburg & Reich 2013:252–256). Unpredicted, uncontrolled behaviour by relatives diagnosed with BPD was found to have a negative effect on family members (Liang et al. 2016:191). Kreger and Mason (2016:251) claim that family members often become isolated as their relative diagnosed with BPD insists that family members cut off their ties with other people. Family members also experienced humiliation. Relatives diagnosed with BPD frequently acted impulsively, resulting in destructive and inappropriate behaviour. Family members of relatives diagnosed with BPD felt judged by their extended family and felt that they were viewed as ineffective in handling their relative’s behaviours.

Ekdahl et al. (2011:69–76) discuss how family members tiptoe around their relative diagnosed with BPD, which often causes strained family relationships, shame and stigma. Family members suffer from the social and emotional stress caused by stigmatisation. As a result, they avoid seeking social support (Park & Park 2014:165–171).

Family members often experience both guilt and shame. They believe that they are the reason for their relative’s problem (Kreger 2009:75). Rivera-Segarra et al. (2014:1e18) state that stigmatisation causes people to feel that they have lost their status; they feel separated and discriminated. Fear of rejection causes family members to begin to withdraw from their social environment, resulting in the loss of their social support system (Sledge et al. 2011:541–544).

Financial strain created by their relatives’ inappropriate behaviours was evident in this study. Family members paid for medical treatment to manage the disorder, drug or alcohol rehabilitation, inpatient psychiatric treatment, along with the damages incurred to themselves and others. Parents of female offspring with BPD experience burden in many areas of their life and have incurred substantial financial expense (Goodman et al. 2011:59–74). Bauer et al. (2012:956–971) indicate that frequent statements by family members of relatives diagnosed with BPD included worries about the burden on other family members, poor cooperation with clinical centres and other institutions by relatives diagnosed with BPD, financial burdens, concerns about the relative’s future and dissatisfaction with the relative’s treatment and rehabilitation.

Marital discord was experienced owing to extra conflict and ongoing stressful challenges created at home. Caring for a person with a personality disorder affects relationships owing to conflict and concern between family members and isolation from friends (Ekdahl et al. 2011:69–76; Goodman et al. 2011:59–74). It is not uncommon for levels of stress to lead to strain in the marriage, resulting in separation, or even divorce (Salters-Pedneault 2017:3).

### Family members experienced a challenging process of adaptation and coping

Family quarrels and fights led to poor, ineffective coping strategies by family members. This resulted in feelings of disempowerment by family members as they lacked the ability to control the circumstances in their family life. Health services were available but were limited and understaffed. There was limited knowledge and information available to educate the family members to effectively cope with their relatives diagnosed with BPD.

Porr (2010:6, 219–220) reports that family members feel powerless to protect the relative diagnosed with BPD from harm and are unable to find effective ways to help. Friends may ostracise a family dealing with a mental illness as if the disorder is as contagious as the ‘flu’ or tuberculosis.

Ineffective coping strategies were related to a lack of communication skills and knowledge by family members of relatives diagnosed with BPD. The lack of awareness of BPD resulted in the health system’s failure to meet the needs of their patients. Some medical practitioners refused to treat patients diagnosed with BPD owing to their lack of knowledge. Porr (2010:6, 17) states that for unknown reasons, relatives diagnosed with BPD will cut off all communication and isolate themselves from family members. Family members learn that programmes and residential facilities do not always teach their relatives diagnosed with BPD how to control their urges.

Ineffective coping skills were often related to a lack of knowledge by family members. This prevented family members from making appropriate choices in assisting their relatives diagnosed with BPD.

### Family members experienced a quest for harmony and integration

Family members wanted to change their approach of caring for their relatives diagnosed with BPD and become reconnected to them. Family members wanted to be self-determined, self-empowered and able to trust, care and support themselves. They were motivated to care in an empowered way.

Family members of relatives diagnosed with BPD in this study experienced that they started to develop a positive approach towards their caregiving roles for relatives diagnosed with BPD. They felt confident and empowered to engage in new experiences, gained inner strength and accelerated their personal growth (Kenny, King & Hall 2014:646–659). Kreger (2009:95–96) claims that family members need to take responsibility for their own development.

Family members were eager to develop a renewed sense of trust and support. They were willing to develop new skills and accept the community’s support. They identified what was helpful. Porr (2010:10) states that there are a few reliable, scientifically based books available on BPD that may be useful for family members.

The family members needed to be self-caring in order to be adequately skilled to provide care for their relatives diagnosed with BPD. Family members learnt to not personalise or defend themselves (Porr 2010:255). Kreger and Mason (2016:91) suggest that family members develop a sense of themselves.

Family members also experienced the need for professional and community support. Until recently, the Australian mental health services did not acknowledge the necessity for the treatment and care of patients diagnosed with BPD. These patients were seen as difficult, troublesome, beyond help and as a result, they were denied service (Lawn & McMahon 2015:235; National Health and Medical Research Council 2012; Rogers & Acton 2012:341–347; Stroud & Parsons 2013:242–253). Family members did not know how to be effective at caring for their relative diagnosed with BPD (Porr 2010:xv).

## Limitations of the study

This study was conducted in a psychiatric hospital. Therefore, the findings may not be applicable to family members with a relative diagnosed with BPD in the community.

## Recommendations

Caring for a relative diagnosed with BPD causes stress and conflict for both the family member and the relative. More support for family members is recommended. This support could be in the form of empowering family members. The results of this study indicated that family members experienced being disempowered because of their lack of knowledge and skills regarding the management of their relatives diagnosed with BPD. This led to the challenging process of adaptation and coping experienced by the family members. Knowledge and skills would empower family members to manage their own mental health more effectively, as well as their relatives diagnosed with BPD. More knowledge about BPD, the understanding and implication of the diagnosis, treatment and care, is needed from mental healthcare professionals and service providers.

## References

[CIT0001] Aguirre B. & Galen G., 2013, *Mindfulness for borderline personality disorder*, New Harbinger Publications Inc, Oakland, CA.

[CIT0002] American Psychiatric Association (APA), 2013, *Diagnostic and statistical manual of mental disorders*, 5th edn., American Psychiatric Publishing, Arlington, VA.

[CIT0003] Bademli K. & Duman Z.C., 2016, ‘Emotions, ideas and experiences of caregivers of patients with schizophrenia about “family to family support program”’, *Archives of Psychiatric Nursing* 30, 329–333. 10.1016/j.apnu.2015.12.002 27256937

[CIT0004] Bailey R.C. & Grenyer B.F.S., 2013, ‘Burden and support needs of carers of persons with borderline personality disorder: A systematic review’, *Harvard Review of Psychiatry* 21, 248–58. 24651557 10.1097/HRP.0b013e3182a75c2c

[CIT0005] Bailey R.C. & Grenyer B.F.S., 2014, ‘Supporting a person with personality disorder: A study of carer burden and well-being’, *Journal of Personality Disorders* 28, 796–809. 10.1521/pedi_2014_28_136 24689763

[CIT0006] Bauer R., Döring A., Schmidt T. & Spießl H., 2012, ‘“Mad or bad?”: Burden on caregivers of patients with personality disorders’, *Journal of Personality Disorders* 26(6), 956–971. 10.1521/pedi.2012.26.6.956 23281679

[CIT0007] Bodner E., Cohen-Fridel S., Mashiah M., Segal M., Grinshpoon A., Fischel T. et al., 2015, ‘The attitudes of psychiatric hospital staff toward hospitalization and treatment of patients with borderline personality disorder’, *BMC Psychiatry* 15(1), 2 10.1186/s12888-014-0380-y 25609479 PMC4307152

[CIT0008] Bowen M., 2013, ‘Borderline personality disorder: Clinician’s accounts of good practice’, *Journal of Psychiatric and Mental Health Nursing* 20(6), 491–498. 10.1111/j.1365-2850.2012.01943.x 22727023

[CIT0009] Brooke S. & Horn N., 2010, ‘The meaning of self-injury and overdosing amongst women fulfilling the diagnostic criteria for borderline personality disorder’, *Psychology and Psychotherapy: Theory, Research and Practice* 83(2), 113–128. 10.1348/147608309X468211 20021731

[CIT0010] Burns N. & Grove S.K., 2013, *The practice of nursing research, conduct, critique, and utilization*, 5th edn., Elsevier Saunders, St Louis, MO.

[CIT0011] Creswell J.W., 2013, *Qualitative inquiry and research design: Choosing among five approaches*, 3rd edn., Sage, Thousand Oaks, CA.

[CIT0012] Creswell J.W., 2014, *Qualitative inquiry and research design: Choosing amongst five traditions*, Sage, Thousand Oaks, CA.

[CIT0013] Crowell S.E., Beauchaine T.P. & Linehan M.M., 2009, ‘A biosocial developmental model of borderline personality: Elaborating and extending Linehan’s theory’, *Psychological Bulletin* 135(3), 495–510. 10.1037/a0015616 19379027 PMC2696274

[CIT0014] De Vos A.S., Strydom H., Fouché C. & Delport C.S.L., 2011, *Research at grass roots for the social sciences and human service professions*, Van Schaik, Pretoria.

[CIT0015] Dhai A. & McQuoid-Mason D.J., 2011, *Bioethics, human rights and health law: Principles and practice*, Juta, Cape Town.

[CIT0016] Dunne E. & Rogers B., 2013, ‘“It’s us that have to deal with it seven days a week”: Carers and borderline personality disorder’, *Community Mental Health Journal* 49(6), 643–648. 10.1007/s10597-012-9556-4 23054157

[CIT0017] Ekdahl S., Idvall E., Samuelsson M. & Perseius K.I., 2011, ‘A life tiptoeing: Being a significant other to persons with Borderline Personality Disorder’, *Archives of Psychiatric Nursing* 25(6), e69–e76. 10.1016/j.apnu.2011.06.005 22114808

[CIT0018] Feigenbaum J., 2010, ‘Self-harm – the solution not the problem: The dialectical behaviour therapy model’, *Psychoanalytic Psychotherapy* 24(2), 115–134. 10.1080/02668731003707873

[CIT0019] Fruzzetti A.E., Crook W., Erikson K.M., Lee J.E. & Worrall J.M., 2009, ‘Emotion regulation’, in O’Donohue W.T. & Fisher J.E. (eds.), *General principles and empirically supported techniques of cognitive behavior therapy*, pp. 272–284, John Wiley & Sons Inc, Hoboken, NJ.

[CIT0020] Goodman M., Patil U., Triebwasser J., Hoffman P., Weinstein Z.A. & New A., 2011, ‘Parental burden associated with borderline personality disorder in female offspring’, *Journal of Personality Disorders* 25(1), 59–74. 10.1521/pedi.2011.25.1.59 21309623

[CIT0021] Gray J.R., Grove S.K. & Sutherland S., 2017, *Burns and Grove’s The Practice of Nursing Research: Appraisal, synthesis, and generation of evidence*, Elsevier, St. Louis, MO.

[CIT0022] Houser J., 2012, *Nursing research reading, using and creating evidence*, Jones & Bartlett Learning, London.

[CIT0023] Kaplan H.I. & Saddock B.J., 2015, *Kaplan & Sadock’s synopsis of psychiatry*, Williams & Wilkins, Baltimore, MD.

[CIT0024] Kay M.L. (2017). A model for the facilitation of mental health of family members who have a relative diagnosed with Borderline Personality Disorder in a psychiatric inpatient unit. D Cur Psychiatric Nursing Thesis, University of Johannesburg, Johannesburg.

[CIT0025] Kenny P., King M.T. & Hall J., 2014, ‘The physical functioning and mental health of informal carers: Evidence of care-giving impacts from an Australian population-based cohort’, *Health and Social Care in the Community* 22, 646–659. 10.1111/hsc.12136 25167764

[CIT0026] Kreger L., 2009, *The essential family guide to borderline personality disorder: New Tools and techniques to stop walking on eggshells*, Simon and Schuster, New York.

[CIT0027] Kreger R. & Mason P.T., 2016, *Stop walking on eggshells*, New Harbinger Publications Inc, Oakland, CA.

[CIT0028] Kvale S. & Brinkman S., 2014, *Learning the craft of qualitative research interviewing*, 3rd edn., Sage, London.

[CIT0029] Lawn S. & McMahon J., 2015, ‘Experiences of family carers of people diagnosed with borderline personality disorder’, *Journal of Psychiatric and Mental Health Nursing* 22, 234–243. 10.1111/jpm.12193 25857849

[CIT0030] Liang X., Guo Q., Luo J., Li F., Ding D., Zhao Q. & Hong Z., 2016, ‘Anxiety and depression symptoms among caregivers of care-recipients with subjective cognitive decline and cognitive impairment’, *BMC Neurology* 16, 191 10.1186/s12883-016-1712-2 27716098 PMC5048476

[CIT0031] Lincoln Y.S. & Guba E.G., 1985, *Naturalistic inquiry*, Sage, London.

[CIT0032] LoBiondo-Wood G. & Haber J., 2014, *Nursing research methods and critical appraisal for evidence-based practice*, Elsevier, St. Louis, MO.

[CIT0033] National Health and Medical Research Council (NHMRC), 2012, *Clinical practice guideline for the management of borderline personality disorder*, National Health and Medical Research Council, Melbourne viewed 06 January 2014, from http://www.nhmrc.gov.au/guidelines/publications/mh25.

[CIT0034] Oxford Dictionaries, 2018a, *Diagnosis*, viewed 28 May 2018, from https://en.oxforddictionaries.com/definition/diagnosis

[CIT0035] Oxford Dictionaries, 2018b, *Family members*, viewed 17 May 2018, from https://en.oxforddictionaries.com/definition/family

[CIT0036] Park S. & Park K.S., 2014, ‘Asian nursing research. Family stigma: A concept analysis’, *Korean Society of Nursing Science* 8(3), 165–171.

[CIT0037] Polit D.F. & Beck C.T., 2017, *Nursing research: Generating and assessing evidence for nursing practice*, Wolters-Kluwer, Philadelphia.

[CIT0038] Porr V., 2010, *Overcoming borderline personality disorder: A family guide for healing and change*, Oxford University Press, New York.

[CIT0039] Rivera-Segarra E., Rivera G., Lopez-Soto R., Crespo-Ramos G. & Marques-Reves D., 2014, ‘Stigmatization experiences among people living with borderline personality disorder in Puerto Rica’, *The Qualitative Report* 19, 1e18.

[CIT0040] Rogers B. & Acton T., 2012, ‘I think we’re all guinea pigs really: A qualitative study of medication and borderline personality disorder’, *Journal of Psychiatric and Mental Health Nursing* 19, 341–347. 10.1111/j.1365-2850.2011.01800.x 22070628

[CIT0041] Salters-Pedneault K., 2017, *How borderline personality disorder can affect a family*, viewed 13 October 2017, from https://www.verywell.com/the-bpd-family-425215

[CIT0042] Sansone R.A. & Sansone L.A., 2013, ‘Responses of mental health clinicians to patients with borderline personality disorder’, *Innovative Clinical Neuroscience* 10(5–6), 39–43. PMID: . 23882440 PMC3719460

[CIT0043] Sledge W.H., Lawless M., Sells D., Wieland M., O’Connell M.J. & Davidson L., 2011, ‘Effectiveness of peer support in reducing re-admission of persons with multiple psychiatric hospitalizations’, *Psychiatric Services* 62, 541–544. 10.1176/ps.62.5.pss6205_0541 21532082

[CIT0044] Stepp S.D., Pilkonis P.A., Yaggi K.E., Morse J.Q. & Feske U., 2009, ‘Interpersonal and emotional experiences of social interactions in borderline personality disorder’, *Journal of Nervous Mental Disorders* 197(7), 484–491. 10.1097/NMD.0b013e3181aad2e7 PMC2757039 19597355

[CIT0045] Stroud J. & Parsons R., 2013, ‘Working with borderline personality disorder: A small-scale qualitative investigation into community psychiatric nurses’ constructs of borderline personality disorder’, *Personal Mental Health* 7(3), 242–253. 10.1002/pmh.1214 24343967

[CIT0046] Wedig M.M., Franenburg F.R. & Reich D.B., 2013, ‘Predictors of suicide threats in patients with borderline personality disorder over 16 years of prospective follow-up’, *Psychiatry Research* 208, 252–256. 10.1016/j.psychres.2013.05.009 23747235 PMC3876888

[CIT0047] Weimand B.M., Hedelin B., Sällström C. & Hall-Lord M.L., 2010, ‘Burden and health in relatives of persons with severe mental illness: A Norwegian cross-sectional study’, *Issues in Mental Health Nursing* 31, 804–815. 10.3109/01612840.2010.520819 21142601

[CIT0048] World Health Organization, 2017, *Mental disorders*, viewed 17 October 2017, from http://www.who.int/mediacentre/factsheets/fs396/en

